# Accuracy of genomic selection for grain yield and agronomic traits in soft red winter wheat

**DOI:** 10.1186/s12863-019-0785-1

**Published:** 2019-11-01

**Authors:** Dennis N. Lozada, R. Esten Mason, Jose Martin Sarinelli, Gina Brown-Guedira

**Affiliations:** 1 Crop, Soil and Environmental Sciences Department, University of Arkansas, Fayetteville, AR 72701 USA; 2 Present Address: Department of Crop and Soil Sciences, Washington State University, Pullman, WA 99164 USA; 3 GDM Seeds Inc, Marion, AR 72364 USA; 4 Department of Crop and Soil Sciences, North Carolina State University, Raleigh, NC 27607 USA; 5 USDA-ARS Plant Science Research and Department of Crop and Soil Sciences, North Carolina State University, Raleigh, NC 27607 USA

**Keywords:** Agronomic traits, Genomic selection, Grain yield, Ridge regression best linear unbiased prediction, Soft red winter wheat, Yield components

## Abstract

**Background:**

Genomic selection has the potential to increase genetic gains by using molecular markers as predictors of breeding values of individuals. This study evaluated the accuracy of predictions for grain yield, heading date, plant height, and yield components in soft red winter wheat under different prediction scenarios. Response to selection for grain yield was also compared across different selection strategies- phenotypic, marker-based, genomic, combination of phenotypic and genomic, and random selections.

**Results:**

Genomic selection was implemented through a ridge regression best linear unbiased prediction model in two scenarios- cross-validations and independent predictions. Accuracy for cross-validations was assessed using a diverse panel under different marker number, training population size, relatedness between training and validation populations, and inclusion of fixed effect in the model. The population in the first scenario was then trained and used to predict grain yield of biparental populations for independent validations. Using subsets of significant markers from association mapping increased accuracy by 64–70% for grain yield but resulted in lower accuracy for traits with high heritability such as plant height. Increasing size of training population resulted in an increase in accuracy*,* with maximum values reached when ~ 60% of the lines were used as a training panel. Predictions using related subpopulations also resulted in higher accuracies. Inclusion of major growth habit genes as fixed effect in the model caused increase in grain yield accuracy under a cross-validation procedure. Independent predictions resulted in accuracy ranging between − 0.14 and 0.43, dependent on the grouping of site-year data for the training and validation populations. Genomic selection was “superior” to marker-based selection in terms of response to selection for yield. Supplementing phenotypic with genomic selection resulted in approximately 10% gain in response compared to using phenotypic selection alone.

**Conclusions:**

Our results showed the effects of different factors on accuracy for yield and agronomic traits. Among the factors studied, training population size and relatedness between training and validation population had the greatest impact on accuracy. Ultimately, combining phenotypic with genomic selection would be relevant for accelerating genetic gains for yield in winter wheat.

## Background

High-throughput genotyping technologies that generate large sets of DNA marker data at low-cost have accelerated the adoption of genomic selection (GS) in plant breeding programs [1]. GS is a molecular breeding tool that predicts genomic estimated breeding values of individuals with only genotypic information available through prediction models constructed based on a training population with genome-wide marker and phenotypic data available [2]. GS complement traditional breeding strategies and can potentially reduce the need for large-scale phenotyping and accelerate the rate of genetic gain through shorter breeding cycles [3–5].

GS was initially implemented in animal breeding, particularly of cattle [2, 6] and has now been extended to different crops, including rice [7, 8], tomato [9, 10], maize [11], soybean [12], and barley [13]. In soft red winter wheat, GS studies have been conducted for *Fusarium* head blight (FHB) resistance [14], grain yield and stability traits [15], yield, softness equivalence, flour yield [16], grain yield, plant height, heading date, and flour quality traits [17], and normalized difference vegetative index (NDVI) [18]. The performance of GS depends primarily on the prediction accuracy, defined as the Pearson’s correlation between the selection criterion and the true breeding value to select individuals with unknown phenotypes [19]. Factors affecting GS accuracy include gene effects, genetic composition of the training population (TP), level of linkage disequilibrium, marker density, statistical models, number of quantitative trait loci (QTL), relationship between TP and the validation population (VP) or selection candidates, TP size, and trait heritability [19–21].

Muleta et al. [22] recently evaluated the effects of trait architecture, size of TP, and different marker densities on GS accuracies for stripe rust in a diverse collection of spring wheat. The genetic complexity of traits with agricultural and economic importance in wheat, such as grain yield and yield components, limit the power of association mapping in identifying small effect loci [23]. GS can circumvent this problem by implementing genome-wide markers for predictions, and thus can complement association analyses in dissecting the genetic basis of important traits [24, 25]. Currently, there are no reports on the accuracy of GS for a diverse population of soft red winter wheat lines that are adapted to southeastern region of the US. Our objectives were then to (1) evaluate the effects of marker number, TP size, relatedness between TP and validation set, presence of fixed effect in the model, and genetic relatedness on accuracy of GS using cross-validations; (2) validate GS model in two biparental populations related to the TP (independent predictions); and (3) compare phenotypic (PS), genomic (GS), marker-based (MS), and random selection (RS) strategies in terms of response to selection (*R*), as a measure of genetic gain for grain yield.

## Results

### Trait heritability and yield across environments

Broad-sense heritability (*H*^*2*^) of grain yield in different environments used for GS are presented in Table 1. In the training population of diverse soft red winter wheat lines, *H*^*2*^ for the measured traits were 0.48 (grain yield), 0.63 (heading date), 0.47 (kernel weight spike^− 1^), 0.37 (kernel number spike^− 1^), 0.77 (thousand kernel weight), and 0.81 (plant height). Values of *H*^*2*^ for grain yield datasets across the three populations ranged between 0.33 (PA_ALL) and 0.85 (PA_Cluster3), with mean grain yield between 2.82 (NB_NPT) and 5.56 t ha^− 1^ (PA_Cluster3) (Table 1). Within the training population, *H*^*2*^ for grain yield ranged between 0.40 (BLUP14) and 0.80 (BLUP15).

**Table 1:** Heritability and yield across different populations of soft red winter wheat used for genomic selection

Population	No. of lines	Dataset	Environments ^a^	Mean (t ha^− 1^)	Min	Max	*H* ^*2* b^
Training population	239	ABLUP	FAY14, FAY15, KEI15, MAR15, OKL15, NPT15 STU14, ROH15	3.10	0.07	7.14	0.48
		BLUP14	FAY14, STU14	2.91	0.37	6.49	0.40
		BLUP15	FAY15, KEI15, MAR15, OKL15, NPT15, ROH15	3.31	0.07	7.60	0.80
		NBLUP	FAY14, FAY15, KEI15, OKL15	3.32	0.07	7.14	0.61
		SBLUP	MAR14, MAR15, STU14, ROH15	2.88	0.37	5.66	0.60
‘NC-Neuse’ x ‘Bess’ (NB)	100	NB_ALL	FAY15, FAY16, FAY17, NPT16, NPT17	3.63	0.03	7.49	0.70
		NB_FAY	FAY15, FAY16, FAY17	4.38	1.04	7.49	0.70
		NB_NPT	NPT16, NPT17	2.82	0.03	5.91	0.45
‘Pioneer Brand 26R61’ x ‘AGS 2000’ (PA)	156	PA_ALL	FAY12, FAY13, FAY14, GA12, GA13, LA13, MAR13, MAR14, STU13, STU14, TX12, TX13	4.40^c^	1.86	6.25	0.33
		PA_Cluster1	FAY12, STU12, FAY14	4.09	3.34	4.81	0.50
		PA_Cluster2	FAY13, MAR14	4.69	3.34	5.69	0.63
		PA_Cluster3	GA12, GA13	5.56	1.47	7.41	0.85
		PA_Cluster4	MAR13, STU13, TX12, TX13	4.00	2.81	4.98	0.66

^*a*^ Indicate site-years included to calculate BLUP for each dataset used for genomic selection

^b^ Broad-sense heritability, calculated using the formula: *H*^*2*^$=\frac{\sigma_G^2}{\sigma_G^2+{\sigma}_{\frac{GEI}{e}}^2+{\sigma}_{\frac{E}{er}}^2\ }$

^c^ Results adapted from Mason et al. [18]

### Effect of marker number and training population size

**Fig. 1:**
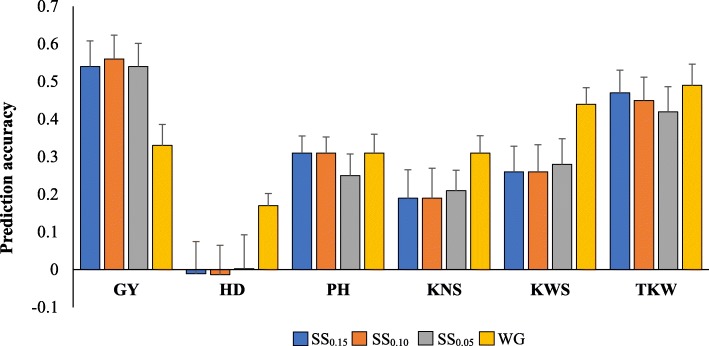
Accuracy for yield and agronomic traits under different marker sets for genomic selection. *GY*- grain yield; *PH*- plant height; *HD*- heading date; *TKW*- thousand kernel weight; *KNS*- kernel number per spike; *KWS*- kernel weight per spike. *SS*_*0.15*_- marker subset based on significance level *P <* 0.15 (~ 820 SNPs); *SS*_*0.10*_*-* marker subset based on significance level *P <* 0.10 (~ 540 SNPs); *SS*_*0.05*_*-* marker subset based on significance level *P <* 0.05 (~ 270 SNPs); *WG*- whole genotype marker data (~ 5600 SNPs). Bars indicate standard errors

Average number of markers used for GS for each subset (SS) were 820 (SS_0.15_), 540 (SS_0.10_), and 270 (SS_0.05_) SNPs. Prediction accuracies for grain yield increased from 0.33 to 0.56 when SS_0.10_ was used for predictions (Fig. 1; Additional file 1: Table S1). Comparable prediction values were observed between the marker subsets, with both SS_0.05_ and SS_0.15_ having similar accuracy (0.54). Using less markers, on the other hand, was not that successful for heading date, in which using SS_0.15_ and SS_0.10_ resulted in negative accuracies (− 0.01), probably resulting from using a smaller number of markers. For plant height, similar accuracies were observed for SS_0.10_, SS_0.15_, and whole genotype data (0.31), whereas using SS_0.05_ resulted in marginal decrease in accuracy (0.31 to 0.25). For the yield components, there was a 14–39% decrease in accuracy when using the marker SS for predictions. Using random SNP marker sets resulted in accuracies between 0.07 (heading date) and 0.46 (thousand kernel weight). Relative to the GWAS-derived markers, using the random SNPs caused a significant (*P* < 0.0001) reduction in prediction accuracies (0.34 vs. 0.55) for grain yield. In contrast, significantly higher prediction accuracies (*P* < 0.05) for random markers were observed for all the other traits except thousand kernel weight. Among the random marker sets, using RM1 (820 random SNPs) and RM3 (270 random SNPs) resulted in similar prediction accuracy (0.30).

**Fig. 2:**
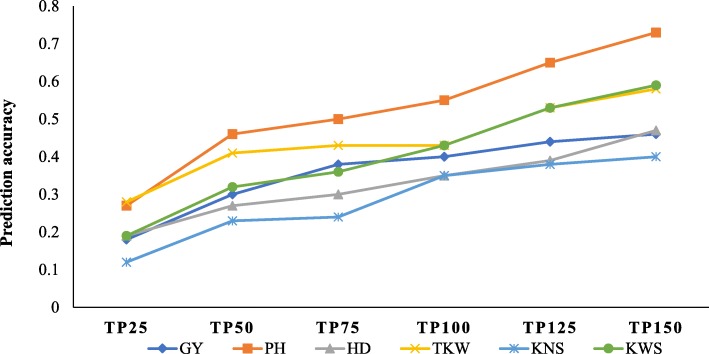
Effect of training population size on accuracy of genomic selection for yield and agronomic traits. *GY*- grain yield; *PH*- plant height; *HD*- heading date; *TKW*- thousand kernel weight; *KNS*- kernel number per spike; *KWS*- kernel weight per spike. Size of validation population (VP) = 60

Increasing training population size resulted in increased accuracy across all the measured traits when validation population size was held constant and reached a maximum at TP150 (Fig. 2; Additional file 1: Table S2). Comparing TP25 with TP150, prediction accuracies increased from 0.18 to 0.46 for grain yield, from 0.27 to 0.73 for plant height (the most heritable trait), and from 0.19 to 0.47 for heading date. For yield components, accuracies increased from 0.12 to 0.40 for kernel number spike^− 1^, 0.19 to 0.59 for kernel weight spike^− 1^, and 0.28 to 0.58 for thousand kernel weight. A minimal increase was observed (between 4.6 and 20.5%) from TP125 to TP150 as accuracy values hit a plateau. No significant differences between the mean accuracy of each training population size across traits were observed for TP100 and TP125 and for TP125 and TP150, whereas accuracy for TP25 was significantly lower *(P <* 0.05) compared to all other training population sizes.

### Effect of population structure and fixed effect in the model

**Fig. 3:**
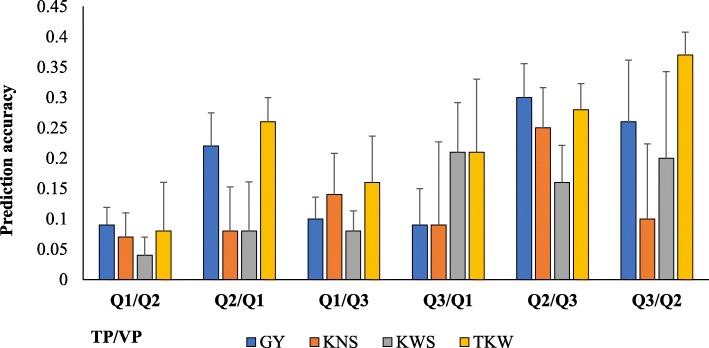
Accuracy for yield and yield components using different subpopulations, *Q* as training (TP) and validation populations (VP). Subpopulations based on STRUCTURE software analyses. Predictions were performed using a constant TP and VP sizes of 50 and 30, respectively under 10-fold cross-validations. *GY*- grain yield; *KNS*- kernel number spike^− 1^; *KWS*- kernel weight spike^− 1^; *TKW*- thousand kernel weight. Bars indicate standard errors

Previous STRUCTURE analyses [26] identified three subpopulations in the training population: *Q1* (*N* = 59 lines), *Q2* (*N* = 54 lines) and *Q3* (*N* = 126 lines), with *Q2* and *Q3* being the most related based on population differentiation coefficient*.* On the average, using *Q2* to predict *Q3* (and vice versa) resulted in the highest accuracies, whereas using *Q1* to predict *Q2* resulted in the lowest accuracies for yield and yield components (Fig. 3; Additional file 1: Table S3). For grain yield, there were no significant differences among GS accuracies when *Q2* was used in predicting *Q3* (and vice versa). Prediction accuracies of 0.09 and 0.10 were observed when *Q1* was used as a training population to predict *Q2* and *Q3,* respectively (Fig. 3). Prediction accuracies of 0.22 and 0.26 were observed when *Q2* was used to predict *Q1* and *Q3*, respectively; whereas using *Q3* to predict *Q1* and *Q2* resulted in prediction accuracies of 0.09 and 0.26. Accuracies for kernel number spike^− 1^ ranged between 0.07 (*Q1*/*Q2;* TP/VP) and 0.25 (*Q3/Q2*). For kernel weight spike^− 1^, accuracies ranged between 0.04 (*Q1/Q2*) and 0.21 (*Q3*/*Q1*) whereas for thousand kernel weight, accuracy values ranged between 0.08 (*Q1*/*Q2*) and 0.37 (*Q3*/*Q2*).

**Fig. 4:**
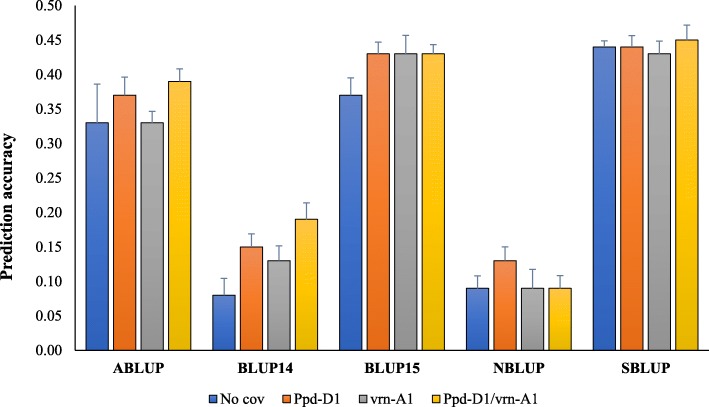
Accuracy for grain yield in the presence or absence (no covariate) of fixed effect in the prediction model. TP size = 144. *ABLUP*- BLUP across all environments; *BLUP14*- BLUP across 2014 environments; *BLUP15*- BLUP across all 2015 environments; *NBLUP*- BLUP across Northern environments; *SBLUP*- BLUP across southern environments. Bars indicate standard errors

In general, GS accuracy for grain yield increased, although marginally, when *Ppd* and *vrn* marker data were as fixed effect in the model (Fig. 4; Additional file 1: Table S4). For the ABLUP dataset, there was an increase in accuracy from 0.33 to 0.37 with the addition of *Ppd-D1*, whereas no increase was observed when *vrn-A1* was added. Using both *Ppd-D1* and *vrn-A1* as fixed effect simultaneously in the model had a greater effect on accuracy for the ABLUP, BLUP14, and BLUP15 datasets compared to using only either locus as a fixed effect. Using *Ppd-D1* increased GS accuracy for all datasets, except for SBLUP. Inclusion of fixed effect in the SBLUP dataset did not lead to significant changes in accuracy.

### Independent predictions using biparental populations

**Fig. 5:**
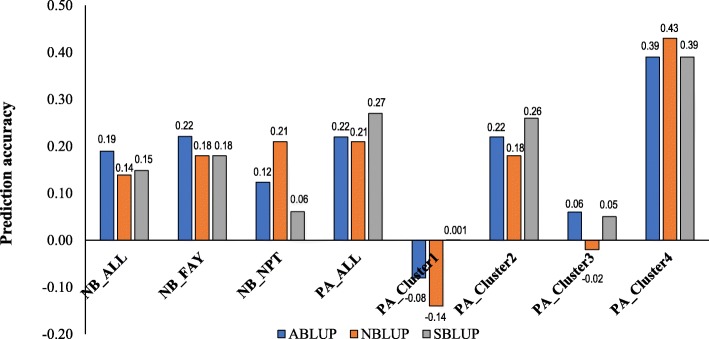
Accuracy for grain yield under independent validations. Training population (*N* = 239 lines; ABLUP, NBLUP, and SBLUP datasets) was used to predict NB (*N* = 100 lines) and PA (*N* = 156 lines) across different site years and clusters. *NB_ALL*- BLUP across all site-years for the NB; *NB_FAY*- BLUP across Fayetteville site-years (FAY15, FAY16, FAY17); *NB_NPT*- BLUP across Newport site-years (NPT16, NPT17); *PA_ALL* represents 12 site-years for the PA; *PA_Cluster1* includes site-years FAY12, STU12, and FAY14; *PA_Cluster2* includes FAY13 and MAR14; *PA_Cluster3* includes GA12 and GA13; *PA_Cluster4* includes TX12, TX13, MAR13, and STU13

Accuracy of the TP to predict two related biparental populations ranged from − 0.14 to 0.43 (Fig. 5; Additional file 1: Table S5). Using NB as a validation population resulted in prediction accuracies ranging from 0.06 to 0.22; whereas using PA as a VP resulted in prediction accuracies between − 0.14 and 0.43. Grouping of site-years in both the training and validation population significantly affected accuracy. For example, PA_Cluster4 was the most predictable (accuracy of 0.40) of the PA site-year groupings, compared to 0.23 in PA_ALL, where all VP site-years were included. Simple matching coefficients reveal a low to moderate similarity between the training population and the PA (0.48) and between the TP and NB (0.45).

### Selection response for grain yield

Response to selection *R* for grain yield was highest for PS + GS (0.34 t ha^− 1^), followed by PS (0.31 t ha^− 1^) and GS (0.21 t ha^− 1^) (Table 2), equal to a 22, 20, and 14% increase above the population mean, respectively. *R* for MS was 0.08 t ha^− 1^ and for RS was 0.01 t ha^− 1^, corresponding to a 4 and 0.63% increase above the population mean. Variance (σ^2^) was highest for RS and MS (both at 0.13) followed by GS (0.12), whereas PS and PS + GS exhibited the lowest σ^2^ at 0.03.

**Table 2:** Response to selection, *R* for grain yield in the training population across different selection strategies

Selection strategy	Grain yield (t ha ^−1^) ± SD	Variance (σ^2^)	Selection differential, *S* ^a^	Response to selection, *R* ^b^	% change relative to PS
GS	3.61 ± 0.34	0.12	0.44	0.21	−32.3
MS	3.34 ± 0.36	0.13	0.17	0.08	− 74.2
PS	3.82 ± 0.16	0.03	0.65	0.31	–
RS	3.19 ± 0.36	0.13	0.02	0.01	−96.8
PS + GS	3.88 ± 0.18	0.03	0.71	0.34	9.70

*GS* genomic selection, *MS* marker-based selection, *PS* phenotypic selection, *PS + GS* phenotypic + genomic selection, *RS* random selection

^a^ S = μ_sel_ - μ_pop_; μ_pop_ = 3.17 t ha^−1^

^b^ Calculated as *R* = *H*^*2*^*S* where *H*^*2*^ is heritability for grain yield based on published value in Lozada et al. [26]; equal to 0.48

## Discussion

The impact of various factors on the accuracy of genomic selection for yield and agronomic traits were evaluated through cross-validations using a diverse panel of soft red winter wheat lines that are adapted to the southeastern region of the US. Effects of marker number, size of TP, relatedness between training and testing set, and the presence of fixed effect in the model were assessed under a ridge regression model (RRBLUP). In another scenario, independent predictions were conducted using the diverse panel to predict grain yield of biparental populations of SRWW. The effects of these parameters in the accuracy of GS are discussed below.

### Accuracy for cross-validations

The number of markers used for GS is crucial to ensure that marker-QTL relationships will be captured for optimum accuracy [19, 27]. Grain yield had higher accuracies when subsets of associated markers were used compared to whole genotype data (0.56 vs 0.33), demonstrating the effectiveness of these marker subsets in capturing marker-QTL linkage disequilibrium (LD) for this trait. Our results agree with a previous study in winter wheat which observed that implementing subsets of associated markers (*P <* 0.05) resulted in the best accuracies for yield [16]. In other crops such as rice [8] and soybean [28], prediction accuracies for grain yield decreased marginally when marker subsets were used. The use of evenly distributed markers was suggested in performing predictions for grain yield and related traits in rice, with the SNP position regarded as the most important factor for accuracy [8]. In this study, selecting the most significant markers (*P* < 0.05) and using them for predictions did not necessarily result in the highest accuracies; in some traits (e.g. for plant height and thousand kernel weight), using the marker subset SS_0.05_ resulted in lower accuracies. For heading date and the yield components, using marker subsets decreased accuracy, irrespective of heritability which suggests that these subsets might not have efficiently captured LD between markers and QTL. Using subsets of markers from association mapping resulted in significantly (*P* < 0.0001) higher accuracies relative to using random SNPs for predicting grain yield, whereas no significant differences was observed for thousand kernel weight. In other traits such as such as plant height and kernel number per spike, nonetheless, using random markers resulted in higher accuracies (Additional file 1: Table S2). Overall, we observed a variable effect of marker number in the accuracy of GS for the evaluated traits, where the genetic architecture of the trait also played a role in determining prediction accuracies. Selecting subsets that can cover the maximum LD between marker and QTL would be advantageous; otherwise for some traits, using whole genotype data will ensure that these relationships will be captured, consequently resulting to better accuracies.

By performing association analyses exclusively on the TP and using the significant loci identified from these as our marker subsets for predictions, we disregarded the “inside trading” effect that results when prediction accuracies are evaluated using QTL identified in the same group of lines [14]. In winter wheat, Arruda et al. [14] previously demonstrated that “inside trading” can lead to inflated values (i.e. ~ 32% overall increase) for GS accuracies for FHB-related traits when significant QTL were treated as fixed effect in the model. We thus showed here that even without “inside trading,” it was still possible improve accuracy for grain yield, which reached a maximum accuracy of 0.56 when SS_0.10_ was used for predictions. In comparison with other studies that performed cross-validations [29, 30], we observed relatively high accuracies for grain yield in the current study, particularly when subsets of markers were used for predictions. One possible reason for this is that we used a population with minimal genetic stratification or structure, hence a smaller number of markers in this case could capture LD relationships between markers and QTL. Previously, it was shown that this panel has only three subpopulations, with no observable clustering of lines based on geographic origin [26]. Moreover, the mean pairwise Chord distance value among the lines was 0.28. These then indicate that genetic relatedness within and among the lines is crucial in obtaining optimal prediction accuracies, particularly when models such as RRBLUP are being implemented.

Increasing training population size increased prediction accuracies across all measured traits but tended to plateau between TP125 and TP150. Increasing number of lines at this point, then, did not give any additional advantage in terms of accuracy. Similarly, in spring wheat, it was recently noted that accuracy values either plateaued at the largest training population size or showed no sign of reaching a plateau depending on the environment and trait [22]. A positive correlation between TP size and accuracy had been observed for biparental and multifamily wheat populations [17, 30], a soybean nested association mapping (NAM) population [28], and elite breeding populations of oats [31]. Increasing TP size increases accuracy by improving the estimation of marker effects [17]. Based on our results for cross-validations, an optimal number of lines (~ 60% of the entire population) should be included in the training panel to achieve improved predictions. Beyond this, increasing TP size might not be longer advantageous for increasing accuracy.

Aside from TP size, the composition and relatedness of the training and validation populations significantly affected prediction accuracy. Using *Q2* to predict *Q3* (and vice versa) for grain yield and component traits gave an 85% advantage over using the less related subgroup *Q1.* These results agree with previous studies that showed higher prediction accuracies for more related populations [17, 28]. In barley, the inclusion of unrelated individuals in a TP reduced accuracy compared to a TP consisting of only highly related individuals [32]. Close relatives share long haplotype and linkage blocks resulting in minimal statistical bias in estimating breeding values and more accurate predictions [33]. In contrast, inconsistent QTL effects of distantly related TP and VP can result in lower prediction accuracies [34]. Relatedness between training and test individuals is thus important for achieving high accuracies; TP should be fully optimized to ensure that it captures most of the genetic relationships with the validation sets.

Including *Ppd-D1* and *vrn-A1* fixed effect in the model resulted in a general increase (although marginally) in the accuracy of grain yield. Mason et al. [18] reported the same trend when using major genes as fixed effect to predict yield using cross-validations in the PA, particularly for site-year groupings with low heritability. The same study also reported that inclusion of multiple loci as fixed effect did not significantly improve prediction accuracies, which could be due to a limited population size used. Likewise, Daetwyler et al. [35] observed that inclusion of marker scores for known rust resistant genes (*Lr34*/*Sr57*/*Yr18*) increased accuracy for rust resistance in diverse wheat germplasm.

Overall, our results demonstrated the effects of different parameters in the accuracy of GS in soft red winter wheat through cross-validations. Training population size and its relatedness to the validation population were the major factors influencing accuracy. Fine-tuning of these parameters would help achieve optimal prediction accuracies towards improving genetic gains in plant breeding programs.

### Accuracy for independent predictions

The goal of GS is to predict the performance of new lines before testing them in the field. With this, we were interested in evaluating prediction accuracies using a TP (*N* = 239 lines) to predict grain yield of biparental populations derived from the cross between parents belonging to the TP. Lower accuracies for grain yield resulted when NB (0.06–0.22) and PA (− 0.14–0.43) were used as VP compared to when predicting through cross-validations, which could be due to low relatedness between the populations. The prediction model used (i.e. RRBLUP) relies mainly on the genetic relationships between training and test populations [36] and hence, its implementation for unrelated lines would not be as successful. In wheat, RRBLUP was also observed to perform poorly when training and testing sets for independent validations were not related [37]. Low accuracies were reported for grain yield, heading date, and test weight using different (unrelated) sets of wheat double haploid and recombinant inbred populations for independent predictions [30]. Highest mean prediction accuracies were observed for Cluster 4, the site-year grouping with highest heritability, consistent with previous results [18]. Within this cluster, using NBLUP dataset which had the highest heritability also resulted in the highest accuracies for grain yield, also demonstrating the influence of heritability in obtaining higher prediction accuracies. The limited relatedness between the TP and the biparental populations (average genetic similarity coefficient of 0.47) could have affected these results. Inclusion of fixed effect may only be effective then in improving accuracies under single population cross-validations or when the training and validation populations are highly related.

Previous GS studies in wheat focused on single population cross-validations of biparental [17, 25] and diversity panels [22], whereas previous reports in other crops such as rice [38] and sugar beet [39] used diverse mapping populations to predict biparental families. Accuracies for grain yield observed here (maximum accuracy of 0.43) demonstrated the potential of using diverse lines to predict complex traits in biparental populations. Similarly, in rice, it was recently shown that prediction models can be trained from a diverse reference population to predict performance among advanced progenies of biparental crosses, with reported prediction accuracies reaching a maximum value of 0.54 [38].

Altogether, we have observed the effects of different factors in the prediction accuracies for yield and agronomic traits. Results from this study could therefore be used as foundation in employing genomic selection approaches in different crops evaluated in multiple environments. For instance, we have observed that a close relatedness between training and test populations would result in optimal accuracies. When implementing genomic selection, breeding programs should therefore create a training population that is able to capture the maximum genetic relationships between the training and validation populations to attain increased accuracies. The size of the training population, particularly for single-population cross-validations, was also observed to affect accuracies, where an increased number of lines is related to improved prediction accuracies. Plant breeding programs should thus build a training population that is “large” enough; nevertheless, caution is warranted as we have observed that there is an optimal training population size and adding more lines might not be advantageous in improving prediction accuracies.

### Response to selection for grain yield

GS is a tool to complement PS in selecting “better” genotypes through estimation of breeding values of individuals [19]. Within the parameters of this study, *R* for GS could only approach the level of PS and therefore showed a lower *R* (− 32% change relative to PS). However, the highest accuracy was observed when GS was coupled with PS, resulting to a 10% increase in *R* compared to using PS alone. Using both phenotype and breeding values for selections, Belamkar et al. [40] observed the feasibility of selecting higher yielding lines to advance in the next season in a winter wheat preliminary yield trial. GS was superior to MS for three significant loci in terms of *R*, whereas using four or more significant QTL for MS might not be beneficial as there would be lower number of individuals being selected. Arruda et al. [14] observed higher selection differentials for GS compared to MS using a maximum of five QTL associated with FHB-related traits in soft red winter wheat. In the same study, it was shown that decreasing selection intensity (i.e. selecting for fewer lines) resulted in an increased selection differential and hence increased *R.* Using simulations in maize double haploid populations, it was demonstrated that across different QTL number and trait heritability, the response to GS was 18–43% greater than response to MS, with an increase in *R* observed as heritability and the number of QTL increased [41]. Ultimately, based on our results, the potential of increasing genetic gains for yield can be achieved through combining GS with PS.

## Conclusions

Different factors were observed to affect accuracy for grain yield and agronomic traits in soft red winter wheat, with training population size and the number of markers having the greatest effects. Inclusion of fixed effect in prediction model increased accuracy for grain yield under single population cross-validations. Ultimately, genomic selection could be exploited further with traditional PS to increase response to selection towards grain yield improvement and increasing genetic gains in plant breeding programs. The effects of the evaluated parameters should be considered when implementing genomic selection not only in winter wheat, but also for other important crops to improve genetic potential and facilitate the process of improvement. Altogether, results could be used as basis in designing and optimizing training population, selecting training and validation populations, and determining the ideal number of markers to be used for genomic selection.

## Methods

### Plant material

The genetic materials used for cross-validations in this study consisted of a panel of soft red winter wheat lines previously utilized for a genome-wide association study ([26]; referred to as training population, TP for the independent validations; *N* = 239 lines). The TP was comprised of genotypes from the SunGrains® (Southeastern University Grains) Breeding Cooperative (www.sungrains.lsu.edu.index.shtml) which included lines from Arkansas, Georgia, Kentucky, Louisiana, North Carolina, and Virginia, among others; and other sources of germplasm adapted to the southeastern region of the US. Two additional biparental populations were used for independent validations: (1) a recombinant inbred line population (referred to as PA; *N* = 156 lines, [42, 43]) derived from a cross between soft red winter wheat cultivars ‘Pioneer Brand 26R61’ and ‘AGS 2000’ (PI612956), and; (2) a double haploid (DH) population (referred to as NB; *N =* 100 lines [44];) derived from a cross between ‘NC-Neuse’ (PI633037 [45];) and ‘Bess’ (PI 642794 [46];).

### Genotypic data

The TP and PA were genotyped using the Illumina® 9 K single nucleotide polymorphism (SNP) chip [47] whereas NB was genotyped with the 90 K *iSelect* assays [48] at the USDA-ARS Eastern Regional Genotyping Laboratory in Raleigh, NC. After filtering and quality control, 5661, 1188, and 2780 SNP markers remained for the training population, NB, and PA, respectively. A total of 1089 and 1632 common SNP markers were used for independent validation with the NB and PA as VP, respectively. Imputation for missing data was done using the expected maximization algorithm [29] and implemented through the package ‘rrBLUP’ [49] in R [50]. Genotype data were converted into a numeric format for GS using the ‘GAPIT’ package [51] in R.

### Phenotypic data

Collection and analyses of the phenotypic data were described previously in [26]. Briefly, data consisted of BLUP values derived from adjusted means evaluated based on an augmented design. Adjusted (least square) means for each genotype were estimated using a restricted maximum likelihood (REML) approach using the PROC MIXED function in SAS v.9.4 [52]. The model used for calculating the adjusted means was Y_ijk_ = μ + Entry_j_ + Loc_i_ + Entry_j_ x Loc_i_ + Block_k_(Loc_i_) + ε_ijkl_,

where Y is the trait of interest; μ is the mean effect; Block_i_ is the effect of the *i*th block; Entry_j_ corresponds to the un-replicated genotypes; Loc_i_ is the effect of the *i*th location; Entry_j_ x Loc_i_ is the effect of genotype-by-environment interactions; Block_k_(Loc_i_) is the effect of blocks nested within environments; and ε is the standard normal errors.

Measured traits included grain yield, plant height, heading date, kernel number spike^− 1^, kernel weight spike^− 1^, and thousand kernel weight were collected in eight environments in Arkansas and Oklahoma, U.S. between 2014 and 2015 planting seasons. Collection and analyses of the phenotypic data for the PA were described previously [18, 42]. The PA was grown in three growing seasons (2012–2014) over twelve site-years in Arkansas (Fayetteville (FAY12, FAY13, FAY14); Marianna (MAR13, MAR14), and Stuttgart (STU13; STU14); and Georgia (Plains, GA; GA12, GA13), Louisiana (Baton Rouge, LA; LA13), and Texas (Farmersville, TX; TX12, TX13) in a randomized complete block design with two replications per site-year. Site-year groupings based from previous site-regression analyses [39] were used for PA as validation population for GS.

Grain yield data for NB was collected in five environments, including in Fayetteville (AR) during seasons 2015, 2016 and 2017 (FAY15, FAY16, and FAY17), and Newport (AR) during 2016 and 2017 (NPT16 and NPT17) in a randomized complete block design with two replications per site year except for FAY15 that had only one replication. Grain yield was recorded by harvesting whole plots, weighing the grains, and adjusting for 13% moisture. BLUP across all locations (NB_ALL), across Fayetteville (NB_FAY) and Newport (NB_NPT) were used for NB as VP dataset for genomic prediction.

Broad sense heritability (*H*^*2*^) was calculated by using the formula: ${H}^2=\frac{\sigma_G^2}{\sigma_G^2+{\sigma}_{\frac{GEI}{e}}^2+{\sigma}_{\frac{E}{er}}^2\ }$, where ${\sigma}_G^2$, ${\sigma}_{GEI}^2$, and ${\sigma}_E^2$ are variances due to genotype, genotype-by-environment, and error, respectively; *e* and *r* are the number of environments and replications. Genotype, environment, and genotype by environment interactions were considered as random effects. Variance components were estimated through PROC Mixed in SAS v 9.4.

### Genomic selection model

Ridge regression best linear unbiased prediction (RRBLUP) model was used for genomic selection (GS) through the ‘rrBLUP’ package [49] in R. RRBLUP considers additive marker effects and is based on the infinitesimal model with all markers sharing a common variance and all effects are shrunken toward zero but allows for markers to have uneven effects [2, 14, 53, 54]. ‘rrBLUP’ uses the function ‘mixed.solve’ which fits any mixed model of the form:

$$ \mathbf{y}=\mathbf{X}\boldsymbol{\upbeta } +\mathbf{Zu}+\varepsilon $$

$$ \mathbf{u}\sim \mathrm{N}\left(0,\mathbf{K}{\sigma^2}_u\right), $$

where **X** is a full-rank design matrix for the fixed effects, **β**; **Z** is the design matrix for the random effects **u**, **K** is a positive semidefinite covariance matrix, obtained from markers using ‘A.mat’ which is an additive relation matrix function; residuals are normal with a mean of zero, with constant variance and **u** and ε being statistically independent [49].

### Genomic selection scenarios

Two GS scenarios were evaluated in this study: (1) a standard single population cross-validation scheme where the effects of different factors such as marker number, size of the TP, relatedness between TP and VP, and fixed effect on accuracy were evaluated; and (2) independent predictions, where the population in GS scenario 1 was used as a TP to predict grain yield in NB and PA.

### Different factors affecting genomic selection accuracy

#### Number of markers and size of the training population

Subsets of markers with varying levels of significance, namely, subset SS_0.15_ (*P <* 0.15), SS_0.10_ (*P <* 0.10), and SS_0.05_ (*P <* 0.05) derived from genome-wide association analysis were used to perform predictions to examine the effects of marker number on GS accuracy. To determine the marker subsets, a total of 10 different TP (*N* = 219 lines) and VP (*N =* 20 lines) sets were generated, and an independent association analyses using the GAPIT package [51] in R under a kinship-principal component *(K-PC*) model (with number of PC = 3) was performed with each TP and the ABLUP dataset. This was done to prevent “inside trading” effect, which occurs when prediction accuracies are evaluated using QTL that were previously identified in the same group of lines, potentially resulting to overestimated accuracies [14]. Whole genotype data were filtered for *p*-values corresponding to marker SS_0.15_, SS_0.10_, and SS_0.05_ from each cycle of GWAS. Mean accuracy for each round of GWAS-GS (total of 10 cycles) for each marker subset was recorded. Model performance using marker sets chosen at random was also evaluated, wherein three different sets corresponding to the average number of markers for SS_0.15_, SS_0.10_, and SS_0.05_ (i.e. 820, 540, and 270 random SNP markers, respectively) were used for predictions.

To test the effect of training population size on the accuracy for the evaluated traits, 50 different subsets of 25, 50, 75, 100, 125, and 150 lines were sampled as TP at a constant VP size of 60. Mean accuracy for each TP size was recorded.

#### Relatedness between training and validation population and fixed effect in the model

The effects of relatedness between the training and validation population were evaluated by grouping the lines based on corresponding membership coefficient, *Q* values derived from STRUCTURE [26] and performing predictions where each subpopulation was used to predict the grain yield and component traits of other subgroups. Given that there was an uneven number of lines belonging to each of the subgroups, a subset of 50 and 30 lines were used as TP and VP, respectively, to perform predictions. Genotypes for major genes including growth habit genes, namely photoperiod (*Ppd-D1*) and vernalization requirement (*vrn-A1*) were included in the model as fixed effect, either individually or in combination. GS accuracies with or without the presence of the fixed effect were compared under 10-fold cross-validations for TP size = 144 lines under different datasets- BLUP for all environments (ABLUP), BLUP for 2014 site-years (BLUP14), BLUP for 2015 site-years (BLUP15), BLUP for northern environments (Fayetteville and Keiser, AR; Okmulgee, OK; NBLUP) and BLUP for southern environments (Marianna, Stuttgart, and Rohwer, AR; SBLUP).

#### Independent validation of genomic selection model using biparental populations

The TP (*N* = 239 lines) was used to predict grain yield in the PA (*N* = 157 lines) and NB (*N =* 100 lines) biparental populations using RRBLUP model. Datasets used for the training set were BLUP across all environments (ABLUP), across northern (NBLUP) and southern locations (SBLUP). Simple matching coefficients between the training and validation populations were calculated using the nominal clustering ‘nomclust’ package and simple matching ‘sm’ function in R to evaluate relatedness between the training and validation populations.

#### Response to selection for grain yield

Response to selection, *R* for mean grain yield across eight site-years was calculated using the formula *R* = *H*^2^*S* [55], where *H*^*2*^ is the heritability for grain yield previously reported by Lozada et al. [26], equal to 0.48; and *S* is the selection differential calculated as the difference between the population mean and mean of population with selection, S = μ_S_ – μ_P,_ under a selection intensity of 10% (i.e. selecting the top 25 lines based on average grain yield and genomic estimated breeding values across all environments, 2014, and 2015 site-years). Selection strategies included phenotypic selection (PS), marker-based selection (MS), genomic selection (GS), random selection (RS), and a combination of PS and GS (PS + GS). Mean for grain yield under PS (μ_PS_) was calculated based on the top 25 highest yielding lines; μ_MS_ was equal to the mean grain yield of the lines having the favorable alleles for three loci, *wsnp_Ex_c2723_5047696* (3B), *wsnp_Ex_c13849_21698240* (4B), and *wsnp_Ex_c48922_53681502* (4B), previously identified to be significantly associated with grain yield in the TP [26]; μ_GS_ was equal to the mean of lines having the highest estimated breeding values (top 25 lines) in 10 different rounds of GS under a 10-fold cross-validation in RRBLUP, with TP size =144 lines; μ_RS_ was computed based on a function to generate 25 random selections, 10 different times and calculating the mean for these selections; μ_GS + PS_ was equal to the mean of the lines with the highest grain yield and estimated breeding values.

## Supplementary information

**Additional file 1: Table S1.** Accuracy of genomic selection for measured traits across different training population sizes at a constant validation population size (*N* = 60 lines). **Table S2.** Accuracy of genomic selection across different marker subsets (SS) from association mapping using BLUP across all environments (ABLUP) dataset. **Table S3.** Accuracy of genomic selection for grain yield and yield components using inferred subgroups *Q* from STRUCTURE analyses. **Table S4.** Accuracy using fixed effect (*Ppd-D1* and *vrn-A1*) in genomic selection model for grain yield in soft red winter wheat. **Table S5.** Accuracy of genomic selection for grain yield using the “NC-Neuse-Bess’ and “Pioneer 26R61-AGS2000′ mapping populations as validation sets.

## Data Availability

The datasets used and/or analyzed in the current study are available from the corresponding author on reasonable request.

## Abbreviations

GS: Genomic selection
GY: Grain yield
KNS: Kernel number spike^− 1^
KWS: Kernel weight spike^− 1^
MS: Marker- based selection
NB: NC-Neuse’ x ‘Bess’ biparental mapping population
PA: Pioneer Brand 26R61’ x ‘AGS 2000′ biparental mapping population
PS: Phenotypic selection
QTL: Quantitative trait loci
RS: Random selection
TKW: Thousand kernel weight
TP: Training population
VP: Validation population

## References

[1] Patel DA, Zander M, Dalton-Morgan J, Batley J, Batley J (2015). Advances in plant genotyping: where the future will take us. Plant genotyping: methods and protocols.

[2] Meuwissen THE, Hayes BJ, Goddard ME (2001). Prediction of total genetic value using genome-wide dense marker maps. Genetics.

[3] Heffner EL, Lorenz AJ, Jannink JL, Sorrells ME (2010). Plant breeding with genomic selection: gain per unit time and cost. Crop Sci.

[4] Muranty H, Troggio M, Sadok IB, Al Rifaï M, Auwerkerken A, Banchi E, Velasco R, Stevanato P, Van De Weg WE, Di Guardo M, Kumar S, Laurens F, Bink M (2015). Accuracy and responses of genomic selection on key traits in apple breeding. Hortic Res.

[5] Nakaya A, Isobe SN (2012). Will genomic selection be a practical method for plant breeding?. Ann Bot.

[6] Hayes BJ, Bowman PJ, Chamberlain AC, Verbyla K, Goddard ME (2009). Accuracy of genomic breeding values in multi-breed dairy cattle populations. Genet Sel Evol.

[7] Onogi A, Watanabe M, Mochizuki T, Hayashi T, Nakagawa H, Hasegawa T, Iwata H (2016). Toward integration of genomic selection with crop modelling: the development of an integrated approach to predicting rice heading dates. Theor Appl Genet.

[8] Spindel J, Begum H, Akdemir D, Virk P, Collard B, Redona E, Atlin G, Jannink JL, McCouch SR (2015). Genomic selection and association mapping in Rice (Oryza sativa): effect of trait genetic architecture, training population composition, marker number and statistical model on accuracy of rice genomic selection in elite, tropical rice breeding lines. PLoS Genet.

[9] Duangjit J, Causse M, Sauvage C (2016). Efficiency of genomic selection for tomato fruit quality. Mol Breed.

[10] Hernández-Bautista A, Lobato-Ortiz R, García-Zavala JJ, Parra-Gómez MA, Cadeza-Espinosa M, Canela-Doñan D, Cruz-Izquierdo S, Chávez-Servia JL (2016). Implications of genomic selection for obtaining F2:3 families of tomato. Sci Hortic.

[11] Zhao Y, Gowda M, Liu W, Würschum T, Maurer HP, Longin FH, Ranc N, Reif JC (2012). Accuracy of genomic selection in European maize elite breeding populations. Theor Appl Genet.

[12] Bao Y, Vuong T, Meinhardt C, Tiffin P, Denny R, Chen S, Nguyen HT, Orf JH, Young ND. Potential of association mapping and genomic selection to explore PI 88788 derived soybean cyst nematode resistance. Plant Genome. 2014;7(3):1-13.

[13] Lorenzana RE, Bernardo R (2009). Accuracy of genotypic value predictions for marker-based selection in biparental plant populations. Theor Appl Genet.

[14] Arruda MP, Lipka AE, Brown PJ, Krill AM, Thurber C, Brown-Guedira G, Dong Y, Foresman BJ, Kolb FL (2016). Comparing genomic selection and marker-assisted selection for Fusarium head blight resistance in wheat (*Triticum aestivum* L.). Mol Breed.

[15] Huang M, Cabrera A, Hoffstetter A, Griffey C, Van Sanford D, Costa J, McKendry A, Chao S, Sneller C (2016). Genomic selection for wheat traits and trait stability. Theor Appl Genet.

[16] Hoffstetter A, Cabrera A, Huang M, Sneller C (2016). Optimizing training population data and validation of genomic selection for economic traits in soft winter wheat. G3: genes, genomes. Genetics.

[17] Heffner EL, Jannink JL, Sorrells ME (2011). Genomic selection accuracy using multifamily prediction models in a wheat breeding program. Plant Genome..

[18] Mason RE, Addison CK, Babar A, Acuna A, Lozada DN, Subramanian N, Arguello MN, Miller RG, Brown-Guedira G (2017). Diagnostic markers for vernalization and photoperiod loci improve genomic selection for grain yield and spectral reflectance in wheat. Crop Sci.

[19] Desta ZA, Ortiz R (2014). Genomic selection: genome-wide prediction in plant improvement. Trends Plant Sci.

[20] Rutkoski J, Singh RP, Huerta-Espino J, Bhavani S, Poland J, Jannink JL, Sorrells M (2015). Efficient use of historical data for genomic selection: a case study of stem rust resistance in wheat. Plant Genome..

[21] Zhong S, Dekkers JCM, Fernando RL, Jannink JL (2009). Factors affecting accuracy from genomic selection in populations derived from multiple inbred lines: a barley case study. Genetics.

[22] Muleta KT, Bulli P, Zhang Z, Chen X, Pumphrey M. Unlocking diversity in germplasm collections via genomic selection: a case study based on quantitative adult plant resistance to stripe rust in spring wheat. Plant Genome. 2017;10(3):1-15. 10.3835/plantgenome2016.12.0124 29293811

[23] Korte A, Farlow A (2013). The advantages and limitations of trait analysis with GWAS: a review. Plant Methods.

[24] Mirdita V, He S, Zhao Y, Korzun V, Bothe R, Ebmeyer E, Reif JC, Jiang Y (2015). Potential and limits of whole genome prediction of resistance to Fusarium head blight and Septoria tritici blotch in a vast central European elite winter wheat population. Theor Appl Genet.

[25] Bentley AR, Scutari M, Gosman N, Faure S, Bedford F, Howell P, Cockram J, Rose GA, Barber T, Irigoyen J, Horsnell R, Pumfrey C, Winnie E, Schacht J, Beauchêne K, Praud S, Greenland A, Balding D, Mackay IJ (2014). Applying association mapping and genomic selection to the dissection of key traits in elite European wheat. Theor Appl Genet.

[26] Lozada DN, Mason RE, Babar MA, Carver BF, Brown-Guedira G, Merrill K, Arguello MN, Acuna A, Vieira L, Holder A, Addison C, Moon DE, Miller RG, Dreisigacker S (2017). Association mapping reveals loci associated with multiple traits that affect grain yield and adaptation in soft winter wheat. Euphytica.

[27] Heffner EL, Sorrells ME, Jannink JL (2009). Genomic selection for crop improvement. Crop Sci.

[28] Xavier A, Muir WM, Rainey KM (2016). Assessing predictive properties of genome-wide selection in soybeans. G3: genes, genomes. Genetics.

[29] Poland J, Endelman J, Dawson J, Rutkoski J, Wu S, Manes Y, Dreisigacker S, Crossa J, Sánchez-Villeda H, Sorrells M, Jannink JL (2012). Genomic selection in wheat breeding using genotyping-by-sequencing. Plant Genome.

[30] Charmet G, Storlie E, Oury FX, Laurent V, Beghin D, Chevarin L, Lapierre A, Perretant MR, Rolland B, Heumez E, Duchalais L, Goudemand E, Bordes J, Robert O (2014). Genome-wide prediction of three important traits in bread wheat. Mol Breed.

[31] Asoro FG, Newell MA, Beavis WD, Scott MP, Jannink JL (2011). Accuracy and training population design for genomic selection on quantitative traits in elite north American oats. Plant Genome..

[32] Lorenz AJ, Smith KP (2015). Adding genetically distant individuals to training populations reduces genomic prediction accuracy in barley. Crop Sci.

[33] Hickey JM, Dreisigacker S, Crossa J, Hearne S, Babu R, Prasanna BM, Grondona M, Zambelli A, Windhausen VS, Mathews K, Gorjanc G (2014). Evaluation of genomic selection training population designs and genotyping strategies in plant breeding programs using simulation. Crop Sci.

[34] Bassi FM, Bentley AR, Charmet G, Ortiz R, Crossa J (2016). Breeding schemes for the implementation of genomic selection in wheat (Triticum spp.). Plant Sci.

[35] Daetwyler HD, Bansal UK, Bariana HS, Hayden MJ, Hayes BJ (2014). Genomic prediction for rust resistance in diverse wheat landraces. Theor Appl Genet.

[36] Lorenz A, Nice L. Training Population Design and Resource Allocation for Genomic Selection in Plant Breeding. In: Varshney R, Roorkiwal M, Sorrells ME, editors. Genomic Selection for Crop Improvement: New Molecular Breeding Strategies for Crop Improvement. Switzerland: Springer; 2017. p. 13–22.

[37] Thavamanikumar S, Dolferus R, Thumma BR (2015). Comparison of genomic selection models to predict flowering time and spike grain number in two hexaploid wheat doubled haploid populations. G3: genes, genomes. Genetics.

[38] Hassen MB, Cao TV, Bartholomé J, Orasen G, Colombi C, Rakotomalala J, Razafinimpiasa L, Bertone C, Biselli C, Volante A, Desiderio F, Jacquin L, Vale G, Ahmadi N (2018). Rice diversity panel provides accurate genomic predictions for complex traits in the progenies of biparental crosses involving members of the panel. Theor Appl Genet.

[39] Würschum T, Reif JC, Kraft T, Janssen G, Zhao Y (2013). Genomic selection in sugar beet breeding populations. BMC Genet.

[40] Belamkar V, Guttieri MJ, Hussain W, Jarquín D, El-basyoni I, Poland J, Lorenz AJ, Baenziger PS (2018). Genomic selection in preliminary yield trials in a winter wheat breeding program. G3: genes, genomes. Genetics.

[41] Bernardo R, Yu J (2007). Prospects for genomewide selection for quantitative traits in maize. Crop Sci.

[42] Addison CK, Mason RE, Brown-Guedira G, Guedira M, Hao Y, Miller RG, Subramanian N, Lozada DN, Acuna A, Arguello MN, Johnson JW, Ibrahim AMH, Sutton R, Harrison SA (2016). QTL and major genes influencing grain yield potential in soft red winter wheat adapted to the southern United States. Euphytica.

[43] Hao Y, Chen Z, Wang Y, Bland D, Buck J, Brown-Guedira G, Johnson J (2011). Characterization of a major QTL for adult plant resistance to stripe rust in US soft red winter wheat. Theor Appl Genet.

[44] Petersen S, Lyerly JH, McKendry AL, Islam MS, Brown-Guedira G, Cowger C, Dong Y, Murphy JP (2017). Validation of Fusarium head blight resistance QTL in US winter wheat. Crop Sci.

[45] Murphy JP, Navarro RA, Leath S, Bowman DT, Weisz PR, Ambrose LG (2004). Registration of “NC-Neuse” wheat. Crop Sci.

[46] McKendry AL, Tague DN, Wright RL, Tremain JA (2007). Registration of ‘Bess’ wheat. J plant Regist.

[47] Cavanagh CR, Chao S, Wang S, Huang BE, Stephen S, Kiani S (2013). Genome-wide comparative diversity uncovers multiple targets of selection for improvement in hexaploid wheat landraces and cultivars. Proc Natl Acad Sci.

[48] Wang S, Wong D, Forrest K, Allen A, Chao S, Huang BE (2014). Characterization of polyploid wheat genomic diversity using a high-density 90 000 single nucleotide polymorphism array. Plant Biotechnol J.

[49] Endelman JB (2011). Ridge regression and other kernels for genomic selection with R package rrBLUP. Plant Genome..

[50] R Development Core Team. R: A language and environment for statistical computing. Vienna: R Foundation for Statistical Computing. http://www.R-project.org/.

[51] Lipka AE, Tian F, Wang Q, Peiffer J, Li M, Bradbury PJ, Gore MA, Buckler ES, Zhang Z (2012). GAPIT: genome association and prediction integrated tool. Bioinformatics.

[52] SAS Institute (2011). SAS system options: reference.

[53] Heffner EL, Jannink JL, Iwata H, Souza E, Sorrells ME (2011). Genomic selection accuracy for grain quality traits in biparental wheat populations. Crop Sci.

[54] He S, Schulthess AW, Mirdita V, Zhao Y, Korzun V, Bothe R, Ebmeyer E, Reif JC, Jiang Y (2016). Genomic selection in a commercial winter wheat population. Theor Appl Genet.

[55] Falconer DS, Mackay TF (1996). Introduction to Quantitative Genetics. Pearson Education.

