# Supplementary material for: Accuracy of genomic selection for grain yield and agronomic traits in soft red winter wheat
Source: BMC Genet. 2019 Nov 1;20:82. doi: 10.1186/s12863-019-0785-1 (PMC6823964; doi:10.1186/s12863-019-0785-1)
Supplement: Supplementary file 1 — Additional file 1: Table S1. Accuracy of genomic selection for measured traits across different training population sizes at a constant validation population size (N = 60 lines). Table S2. Accuracy of genomic selection across different marker subsets (SS) from association mapping using BLUP across all environments (ABLUP) dataset. Table S3. Accuracy of genomic selection for grain yield and yield components using inferred subgroups Q from STRUCTURE analyses. Table S4. Accuracy using fixed effect (Ppd-D1 and vrn-A1) in genomic selection model for grain yield in soft red winter wheat. Table S5. Accuracy of genomic selection for grain yield using the “NC-Neuse-Bess’ and “Pioneer 26R61-AGS2000′ mapping populations as validation sets. [file 12863_2019_785_MOESM1_ESM.docx]

**Accuracy of genomic selection for grain yield and agronomic traits in soft red winter wheat**

Dennis N. Lozada^a,1*^, R. Esten Mason^a^, Jose Martin Sarinelli^b,2^, and Gina Brown-Guedira^c^

^a^ Crop, Soil and Environmental Sciences Department, University of Arkansas, Fayetteville, AR, USA 72701; ^b^ Department of Crop Science, North Carolina State University, Raleigh, NC, USA 27607; ^c^ USDA-ARS Plant Science Research and Department of Crop and Soil Sciences, North Carolina State University, Raleigh, NC, USA 27607; ^*^Corresponding author; e-mail address: dblozada@email.uark.edu

^1^ Current address: Department of Crop and Soil Sciences, Washington State University, Pullman, WA, USA 99164; ^2^GDM Seeds Inc, Marion, AR, USA 72364

| **Table S1.** Supplementary Table S1. Accuracy of genomic selection for measured traits across different training population sizes at a constant validation population size (*N*=60 lines). | | | | | | |
| --- | --- | --- | --- | --- | --- | --- |
|  | GY | PH | HD | TKW | KNS | KWS |
| TP25 | 0.18 | 0.27 | 0.19 | 0.28 | 0.12 | 0.19 |
| TP50 | 0.30 | 0.46 | 0.27 | 0.41 | 0.23 | 0.32 |
| TP75 | 0.38 | 0.5 | 0.3 | 0.43 | 0.24 | 0.36 |
| TP100 | 0.4 | 0.55 | 0.35 | 0.43 | 0.35 | 0.43 |
| TP125 | 0.44 | 0.65 | 0.39 | 0.53 | 0.38 | 0.53 |
| TP150 | 0.46 | 0.73 | 0.47 | 0.58 | 0.4 | 0.59 |
| *GY*- grain yield; *PH*- plant height; *HD*- heading date; *TKW*- thousand kernel weight; *KNS*- kernel number spike^-1^; *KWS*- kernel weight spike^-1^ | | | | | | |

| **Table S2.** Accuracy of genomic selection across different marker subsets (SS) from association mapping using BLUP across all environments (ABLUP) dataset. | | | | | | | |
| --- | --- | --- | --- | --- | --- | --- | --- |
| Trait | SS_0.15_ | SS_0.10_ | SS_0.05_ | RM1 | RM2 | RM3 | Whole genotype |
| Grain yield | 0.54 | 0.56 | 0.54 | 0.33 | 0.35 | 0.34 | 0.33 |
| Heading date | -0.011 | -0.013 | 0.0025 | 0.09 | 0.07 | 0.10 | 0.17 |
| Plant height | 0.31 | 0.31 | 0.25 | 0.32 | 0.29 | 0.30 | 0.31 |
| Kernel no. spike^-1^ | 0.19 | 0.19 | 0.21 | 0.24 | 0.25 | 0.24 | 0.31 |
| Kernel weight spike^-1^ | 0.26 | 0.26 | 0.28 | 0.36 | 0.34 | 0.37 | 0.44 |
| Thousand kernel weight | 0.47 | 0.45 | 0.42 | 0.44 | 0.43 | 0.46 | 0.49 |
| Random marker set (RM) 1, RM2, and RM3 correspond to a set of randomly selected SNPs based on the average number of markers for SS_0.15_, SS_0.10_, and SS_0.05_, respectively (i.e. equal to 820, 540, and 270 SNP markers). | | | | | | | |

| **Table S3.** Accuracy of genomic selection for grain yield and yield components using inferred subgroups *Q* from STRUCTURE analyses. | | | | | |
| --- | --- | --- | --- | --- | --- |
| TP/VP | Grain yield | Kernel no. spike^-1^ | Kernel weight spike^-1^ | Thousand kernel weight |  |
| Q1/Q2 | 0.09 | 0.07 | 0.04 | 0.08 |  |
| Q2/Q1 | 0.22 | 0.08 | 0.08 | 0.26 |  |
| Q1/Q3 | 0.10 | 0.14 | 0.08 | 0.16 |  |
| Q3/Q1 | 0.09 | 0.09 | 0.21 | 0.21 |  |
| Q2/Q3 | 0.30 | 0.25 | 0.16 | 0.28 |  |
| Q3/Q2 | 0.26 | 0.10 | 0.20 | 0.37 |  |
| *TP*- training population; *VP*- validation population | | | | |  |

| **Table S4.** Accuracy using fixed effect (*Ppd-D1* and *vrn-A1*) in genomic selection model for grain yield in soft red winter wheat. | | | | | |
| --- | --- | --- | --- | --- | --- |
|  | ABLUP | BLUP14 | BLUP15 | NBLUP | SBLUP |
| No fixed effect (No covariate) | 0.33 | 0.08 | 0.37 | 0.09 | 0.44 |
| *Ppd-D1* | 0.37 | 0.15 | 0.43 | 0.13 | 0.44 |
| *vrn-A1* | 0.33 | 0.13 | 0.43 | 0.09 | 0.43 |
| *Ppd-D1/vrn-A1* | 0.39 | 0.19 | 0.43 | 0.09 | 0.45 |
| *ABLUP*- BLUP across all environments; *BLUP14*- BLUP across 2014 site-years; *BLUP15*- BLUP across 2015 site-years; *NBLUP*- BLUP across Northern environments; *SBLUP*- BLUP across southern environments | | | | | |

| **Table S5.** Accuracy of genomic selection for grain yield using the “Neuse-Bess’ and “Pioneer 26R61-AGS2000’ mapping population as validation sets. | | | | | | | | |
| --- | --- | --- | --- | --- | --- | --- | --- | --- |
| Validation population | | | | | | | | |
| Training pop. dataset ^a^ | NB_ALL^b^ | NB_FAY | NB_NPT | PA_ALL^c^ | PA_Cluster1 | PA_Cluster2 | PA_Cluster3 | PA_Cluster4 |
| ABLUP | 0.19 | 0.22 | 0.12 | 0.22 | -0.08 | 0.22 | 0.06 | 0.39 |
| NBLUP | 0.14 | 0.18 | 0.21 | 0.21 | -0.14 | 0.18 | -0.02 | 0.43 |
| SBLUP | 0.15 | 0.18 | 0.06 | 0.27 | 0.001 | 0.26 | 0.05 | 0.39 |
| ^a^ *ABLUP*- BLUP across all environments; *NBLUP*- BLUP across Northern environments; *SBLUP*- BLUP across southern environments  ^b^ *NB*- “Neuse-Bess” double haploid mapping population  ^c^ *PA*- “Pioneer 26R61 x AGS2000” recombinant inbred population | | | | | | | | |
